# Treatment of Patients With Early-Stage Colorectal Cancer: ASCO Resource-Stratified Guideline

**DOI:** 10.1200/JGO.18.00214

**Published:** 2019-02-25

**Authors:** Ainhoa Costas-Chavarri, Govind Nandakumar, Sarah Temin, Gilberto Lopes, Andres Cervantes, Marcia Cruz Correa, Rena Engineer, Chisato Hamashima, Gwo Fuang Ho, Fidel David Huitzil, Mona Malekzadeh Moghani, Ala I. Sharara, Mariana C. Stern, Catherine Teh, Sara E. Vázquez Manjarrez, Azmina Verjee, Rhonda Yantiss, Manish A. Shah

**Affiliations:** ^1^ Rwanda Military Hospital, Kigali, Rwanda; ^2^ Columbia Asia Hospitals, Bangalore, India; ^3^ Weill Cornell Medical College, New York, NY; ^4^ American Society of Clinical Oncology, Alexandria, VA; ^5^ Sylvester Comprehensive Cancer Center, Miami, FL; ^6^ Hospital Clinico Universitario, Valencia, Spain; ^7^ The University of Puerto Rico, San Juan, PR; ^8^ The University of Texas M.D. Anderson Cancer Center, Houston, TX; ^9^ Tata Memorial Centre, Mumbai, India; ^10^ National Cancer Center, Tokyo, Japan; ^11^ University of Malaya, Kuala Lumpur, Malaysia; ^12^ Instituto Nacional de Ciencias Médicas y Nutrición Salvador Zubirán, Mexico City, Mexico; ^13^ Shahid Beheshti University, Tehran, Iran; ^14^ American University of Beirut, Beirut, Lebanon; ^15^ Keck School of Medicine of the University of Southern California, Los Angeles, CA; ^16^ Makati Medical Center, Makati, Philippines; ^17^ Homerton University Hospital National Health Service Foundation Trust, London, United Kingdom; ^18^ Bowel Disease Research Foundation, London, United Kingdom; ^19^ New York-Presbyterian/Weill Cornell Medical Center, New York, NY

## Abstract

**PURPOSE:**

To provide resource-stratified, evidence-based recommendations on the treatment and follow-up of patients with early-stage colorectal cancer.

**METHODS:**

ASCO convened a multidisciplinary, multinational Expert Panel that reviewed existing guidelines and conducted a modified ADAPTE process and a formal consensus process with additional experts for one round of formal ratings.

**RESULTS:**

Existing sets of guidelines from 12 guideline developers were identified and reviewed; adapted recommendations from six guidelines form the evidence base and provide evidence to inform the formal consensus process, which resulted in agreement of 75% or more on all recommendations.

**RECOMMENDATIONS:**

For nonmaximal settings, the recommended treatments for colon cancer stages nonobstructing, I-IIA: in basic and limited, open resection; in enhanced, adequately trained surgeons and laparoscopic or minimally invasive surgery, unless contraindicated. Treatments for IIB-IIC: in basic and limited, open en bloc resection following standard oncologic principles, if not possible, transfer to higher-level facility; in emergency, limit to life-saving procedures; in enhanced, laparoscopic en bloc resection, if not possible, then open. Treatments for obstructing, IIB-IIC: in basic, resection and/or diversion; in limited or enhanced, emergency surgical resection. Treatment for IIB-IIC with left-sided: in enhanced, may place colonic stent. Treatment for T4N0/T3N0 high-risk features or stage II high-risk obstructing: in enhanced, may offer adjuvant chemotherapy. Treatment for rectal cancer cT1N0 and cT2n0: in basic, limited, or enhanced, total mesorectal excision principles. Treatment for cT3n0: in basic and limited, total mesorectal excision, if not, diversion. Treatment for high-risk patients who did not receive neoadjuvant chemotherapy: in basic, limited, or enhanced, may offer adjuvant therapy. Treatment for resectable cT3N0 rectal cancer: in enhanced, base neoadjuvant chemotherapy on preoperative factors. For post-treatment surveillance, a combination of medical history, physical examination, carcinoembryonic antigen testing, imaging, and endoscopy is performed. Frequency depends on setting. Maximal setting recommendations are in the guideline. Additional information can be found at www.asco.org/resource-stratified-guidelines.

**NOTICE:**

It is the view of the American Society of Clinical Oncology that health care providers and health care system decision makers should be guided by the recommendations for the highest stratum of resources available. The guidelines are intended to complement but not replace local guidelines.

## INTRODUCTION

The purpose of this guideline is to provide expert guidance on the treatment and post-treatment follow-up of patients with early-stage colorectal cancer to clinicians, public health leaders, and policymakers in all resource settings. The target population is people with early-stage colorectal cancer (colon cancer stages I-IIIC and rectal cancer stages I-III).

Historically, some of the highest incidence rates have been in so-called more-developed regions, including North America, Australia, New Zealand, Western Europe, Japan, and South Korea. However, approximately 45% of incident colorectal cancers in men and women occur in less developed regions (the definition of which often overlaps with the definition of low- and middle-income countries around the world and represent 9% to 10% of cancers among people in those regions).^1^ Fifty-two percent of deaths resulting from colorectal cancer occur in these less developed regions.

These numbers are increasing around the world (eg, increases in occurrences in some Eastern European countries and Japan, increases in deaths in some South American countries and East Europe ).^2^ Different regions of the world, both among and within countries, differ with respect to access to early detection. (Very few countries outside of high-income countries have mass or even opportunistic screening, and even within regions with mass screening, subpopulations may not have access to screening.) As a result of these disparities, the ASCO Resource-Stratified Guidelines Advisory Group chose colorectal cancer as a priority topic for guideline development.

ASCO has established a process for resource-stratified guidelines, which includes mixed methods of guideline development, adaptation of the clinical practice guidelines of other organizations, and formal expert consensus. This article summarizes the results of that process and presents the practice resource-stratified recommendations, which are based in part on formal expert consensus and adaptation from existing guidelines (see Results section and Appendix Table A1).

In developing resource-stratified guidelines, ASCO has adopted its framework from the four-tier resource setting approach (basic, limited, enhanced, and maximal; Table 1) developed by the Breast Health Global Initiative, and modifications to that framework are based on the Disease Control Priorities 3.^3,4^ The framework emphasizes that variations occur not only between but also within countries with disparities, for example, between rural and urban areas, between areas with basic primary care and areas where more-resourced medical care is not available in the local area but, rather, farther away. ASCO uses an evidence-based approach to inform guideline recommendations.

**TABLE 1:**
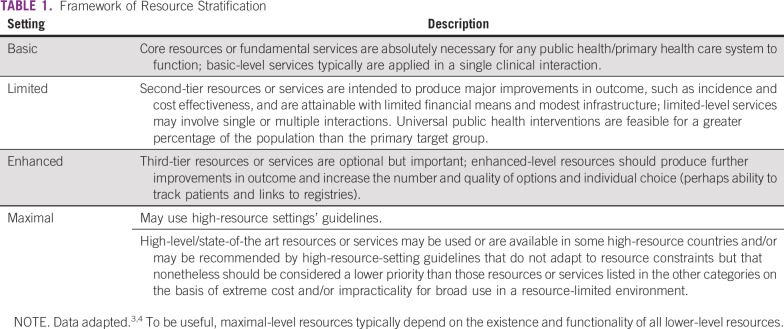
Framework of Resource Stratification

## GUIDELINE QUESTION

This clinical practice guideline addresses the following overarching clinical questions: (1) What is the optimal treatment of patients with colon cancer clinical stages I-IIIC in high-incidence and resource-constrained settings? (2) What is the optimal treatment of patients with rectal cancer stages I-III? (3) What are the optimal strategies for post-treatment surveillance for patients treated for early colorectal cancer?

## METHODS

These recommendations were developed by an Expert Panel with multinational and multidisciplinary representation and with a patient representative and an ASCO guidelines staff member with health research methodology expertise (Appendix Table A2). The Expert Panel met via teleconference and in person and corresponded through e-mail. Based upon the consideration of the evidence, the authors were asked to contribute to the development of the guideline, provide critical review, and finalize the guideline recommendations. Members of the Expert Panel were responsible for reviewing and approving the penultimate version of the guideline, which was then circulated for external review and submitted to a peer-reviewed journal for editorial review and consideration for publication. This guideline was partially informed by ASCO’s modified Delphi Formal Expert Consensus methodology, during which the Expert Panel was supplemented by additional experts recruited to rate their agreement with the drafted recommendations. The entire membership of experts is referred to as the Consensus Panel (the Data Supplement provides a list of members). All ASCO guidelines are ultimately reviewed and approved by the Expert Panel and the ASCO Clinical Practice Guidelines Committee prior to publication.

This guideline adaptation was also informed by the ADAPTE methodology^5^ and consensus processes used together as an alternative to de novo guideline development for this guideline. Adaptation of guidelines is considered by ASCO in selected circumstances when one or more quality guidelines from other organizations already exist on the same topic. The objective of the ADAPTE process is to take advantage of existing guidelines to enhance efficient production, reduce duplication, and promote the local uptake of quality guideline recommendations.

ASCO’s adaptation and formal consensus processes begin with a literature search to identify candidate guidelines for adaptation. Adapted guideline manuscripts are reviewed and approved by the Clinical Practice Guidelines Committee. The review includes two parts: methodological review and content review.^6^ The methodological review was completed by ASCO senior guideline staff (Methodology Supplement). The content review was completed by the ASCO Expert Panel.

The guideline recommendations were crafted, in part, using the Guidelines Into Decision Support (GLIDES) methodology and accompanying BRIDGE-Wiz software.^7^ Detailed information about the methods used to develop this guideline is available in the Methodology Supplement and Data Supplement at www.asco.org/resource-stratified-guidelines.

The ASCO Expert Panel and guidelines staff will work with co-chairs to keep abreast of any substantive updates to the guideline. On the basis of formal review of the emerging literature, ASCO will determine the need to update.

This is the most recent information as of the publication date. For updates, the most recent information, and to submit new evidence, please visit www.asco.org/esource-stratified-guidelines. All funding for the administration of the project was provided by ASCO.

### Guideline Disclaimer

The clinical practice guidelines and other guidance published herein are provided by the American Society of Clinical Oncology, Inc. (“ASCO”) to assist providers in clinical decision making. The information therein should not be relied upon as being complete or accurate, nor should it be considered as inclusive of all proper treatments or methods of care or as a statement of the standard of care. With the rapid development of scientific knowledge, new evidence may emerge between the time information is developed and when it is published or read. The information is not continually updated and may not reflect the most recent evidence. The information addresses only the topics specifically identified therein and is not applicable to other interventions, diseases, or stages of diseases. This information does not mandate any particular course of medical care. Further, the information is not intended to substitute for the independent professional judgment of the treating provider, as the information does not account for individual variation among patients. Recommendations reflect high, moderate or low confidence that the recommendation reflects the net effect of a given course of action. The use of words like “must,” “must not,” “should,” and “should not” indicate that a course of action is recommended or not recommended for either most or many patients, but there is latitude for the treating physician to select other courses of action in individual cases. In all cases, the selected course of action should be considered by the treating provider in the context of treating the individual patient. Use of the information is voluntary. ASCO provides this information on an “as is” basis, and makes no warranty, express or implied, regarding the information. ASCO specifically disclaims any warranties of merchantability or fitness for a particular use or purpose. ASCO assumes no responsibility for any injury or damage to persons or property arising out of or related to any use of this information or for any errors or omissions.

### Guideline and Conflict of Interest

The Expert Panel was assembled in accordance with ASCO’s Conflict of Interest Policy Implementation for Clinical Practice Guidelines (“Policy,” found at http://www.asco.org/rwc). All members of the Expert Panel completed ASCO’s disclosure form, which requires disclosure of financial and other interests, including relationships with commercial entities that are reasonably likely to experience direct regulatory or commercial impact as a result of promulgation of the guideline. Categories for disclosure include employment; leadership; stock or other ownership; honoraria, consulting or advisory role; speaker's bureau; research funding; patents, royalties, other intellectual property; expert testimony; travel, accommodations, expenses; and other relationships. In accordance with the Policy, the majority of the members of the Expert Panel did not disclose any relationships constituting a conflict under the Policy.

## RESULTS

### Literature Search

As part of the systematic literature review, PubMed, Standards and Guidelines Evidence directory (www.cancerview.ca/TreatmentAndSupport/GRCMain/GRCSAGE/GRCSAGESearch), Cochrane Systematic Review, and National Guideline Clearinghouse (NGC) databases were searched for guidelines, systematic reviews, and meta-analyses published between 1966 and 2017 (2012 and 2017 for guidelines). Inclusion criteria identified publications that (1) were on the treatment of early-stage colon and early-stage rectal cancer, (2) developed by multidisciplinary content experts as part of a recognized organizational effort, and (3) published between 1966 and 2017. Searches for cost-effectiveness analyses were also conducted. Articles were excluded from the systematic review if they were (1) meeting abstracts or (2) books, editorials, commentaries, letters, news articles, case reports, or narrative reviews. After initial searches of primary literature, the panel leadership decided to primarily use guidelines to inform expert consensus. Searches for cost-effectiveness analyses were also conducted separately.

A total of 40 guidelines were found in the literature search, and 12 were reviewed in-depth for their currency, content, and methodology (not including ASCO’s endorsement of the Cancer Care Ontario [CCO] follow-up guideline, which was not formally re-reviewed). On the basis of content and methodology reviews (the latter by either ASCO or NGC), the Expert Panel chose six evidence-based guidelines from five public health authorities/guideline developers: Society of American Gastrointestinal and Endoscopic Surgeons (SAGES),^8^ American Society of Colon and Rectal Surgeons (ASCR),^9,10^ UK National Institute for Clinical Excellence (NICE),^11^ European Society for Medical Oncology (ESMO),^12^ and the ASCO endorsement of the CCO guideline.^13^ Appendix Table A1 lists links to the guidelines. The Expert Panel used these guidelines, some literature suggested by the Expert Panel, and clinical experience as guides.

This ASCO guideline reinforces selected recommendations offered in the SAGES evidence-based guidelines for the laparoscopic resection of curable colon and rectal cancer and the National Guideline Alliance/NICE, ESMO, ASCO, and ASCR guidelines. This guideline also acknowledges the effort put forth by the authors and the aforementioned societies to produce evidence-based and/or consensus-based guidelines informing practitioners and institutions who provide guidance on colorectal cancer care and post-treatment follow-up of patients and caregivers.

The identified guidelines were published between 2012 and 2017. If the NGC had not formally reviewed the methodology of a given guideline, ASCO used the AGREE II instrument. The Data Supplement includes an overview of these guidelines, including information on the clinical questions, target populations, development methodology, and key evidence.

### Guidelines on Treatment and Follow-Up of Patients With Early Colon and Rectal Cancer

#### Clinical questions and target population of guidelines adapted by ASCO.

The maximal resource-level settings guidelines adapted in part by ASCO are listed in Appendix Table A1. For the treatment of patients with early colon cancer, the Expert Panel used the SAGES and NICE guidelines as the evidence base. The SAGES guideline, based on a systematic literature review, pertains to patients with colon and rectal cancer eligible for surgery.^8^ The NICE guideline population included adults (defined as 18 years of age and older) with surgically resectable colorectal cancer with newly diagnosed adenocarcinoma of the colon or the rectum or with relapsed adenocarcinoma of the colon or rectum.^11^ The NICE guideline focused on the effectiveness, including cost-effectiveness of laparoscopic surgery (source: NICE Health Technology Appraisal protocol for laparoscopic surgery for the treatment of colorectal cancer).^14^ Selected clinical questions relevant to this ASCO guideline included those on the sequence of treatments, indications for surgery, neoadjuvant chemotherapy (NACT), and adjuvant treatment for patients with stages I-III colon cancer and stages I-III rectal cancer.

For the treatment of patients with early rectal cancer, this guideline used ASCR and ESMO guidelines as well as consensus to inform the recommendations. Clinical questions were not explicitly stated in the ESMO guideline; the studies it included reported on efficacy and toxicity outcomes. The ESMO guideline concerns various potential treatment modalities, including surgery, chemotherapy, and radiation.^12^ ASCR used an organized search to address treatment issues for patients with rectal cancer.^10^ Clinical questions were not explicitly stated, and limited information on the guideline methodology was included within either guideline (ESMO has separate publications on its development methods).

For follow-up, this guideline refers to the ASCO endorsement of the CCO guideline and to the ESMO 2017 rectal cancer guideline.^12,13^ The primary clinical questions concerned benefits, test properties, and adverse events.

#### Summary of guidelines adapted by ASCO: development methodology and key evidence.

The SAGES guideline used a systematic literature review and rated recommendations with Grading of Recommendations Assessment, Development and Evaluation (GRADE). Evidence tables were not included. This guideline received a rating of 50% on the AGREE II from ASCO (Methodology Supplement).

The NICE guideline met the 2013 NGC criteria, which ASCO used as a proxy for quality guidelines. The key evidence includes systematic reviews, meta-analyses, randomized clinical trials (RCTs), and case series, and the assessment methods included GRADE. The developers noted that there were limited high-quality studies comparing multiple interventions.

The ESMO rectal cancer guideline methods included an ISDA-based method for grading evidence and received a 38% AGREE II score from ASCO. The ASCR rectal cancer guideline used GRADE and had a 43% AGREE II score from ASCO. The NGC reviewed the ASCR guideline but found that it did not meet 2013 NGC criteria. However, given the paucity of guidance on the treatment of patients with rectal cancer, the Expert Panel opted to use the ASCR guideline as part of the evidence base. The ASCO guideline endorsement of the CCO guideline on post-treatment follow-up was based on a systematic review of 11 other guidelines that CCO found concurred.

#### Outcomes.

The outcomes/end points in most studies reviewed by the adapted guidelines include efficacy (including overall survival, disease-free survival); quality of life; safety/adverse events; and, in some, cost-effectiveness.

### Results of ASCO Methodological Review

The methodological review of the guidelines was completed by two ASCO guideline staff members using the Rigor of Development subscale of the AGREE II instrument (if the NGC had not previously applied 2013 quality criteria to a guideline). The score for the Rigor of Development domain is calculated by summing the scores across individual items in the domain and standardizing the total score as a proportion of the maximum possible score. Detailed results of the scoring and the AGREE II assessment process for this guideline are available in the Methodology Supplement.

## RECOMMENDATIONS

The recommendations were developed by a multinational, multidisciplinary group of experts using evidence from existing guidelines and clinical experience as a guide. The ASCO Expert Panel underscores that health care practitioners who implement the recommendations presented in this guideline should first identify the available resources in their local and referral facilities and endeavor to provide the highest level of care possible with those resources.

### Clinical Question 1

What is the optimal treatment of patients with colon cancer clinical stages I-IIIC in high-incidence and resource-constrained settings?

The definition of high risk for recurrence in this guideline was defined by the NICE evidence-based review (NICE 2011)^15^; any one of the following characteristics would be considered high risk and generally applies to stage II colon tumors: extramural vascular invasion, grade 3/poorly differentiated tumors, T4 stage/perforation, obstructive tumors, mucinous tumors, examination of fewer than 12 lymph nodes, and tumor budding. Overall, there is limited evidence on the relationship of these characteristics with risk, and evaluation of risk of recurrence in basic and limited settings may be challenging.

Most local and locally advanced colon cancer is treated with surgery. The primary types of surgery are laparoscopic and open resection. The choice of which surgery is performed depends on the tumor characteristics, the local infrastructure, and surgical expertise. Patients with stage III tumors and some patients with high-risk stage II tumors may be eligible for chemotherapy following surgery, where chemotherapy is available. The results of the surgical pathology evaluation may affect the initial clinical staging classification and is one of the main factors driving adjuvant therapy decisions; a second important factor is the availability of chemotherapy itself. Therefore, the resource setting is an important factor in determining the type of treatment.

### Nonobstructing Resectable, Localized Colon Cancer

The primary treatment of patients with colon cancer stages I-IIA is surgery to remove the cancer and draining nodal stations according to standard oncologic principles (Table 2). Advances in surgical technology allow for these aims to be achieved with less-invasive techniques. In basic and limited settings, the overarching recommendation is that surgeons perform open resection and in enhanced and maximal settings, perform minimally invasive surgery. Contraindications to laparoscopy may include whether a patient has a distended bowel; advanced disease, if the procedure cannot achieve an R0 resection; and/or an inability to tolerate pneumoperitoneum. Complete R0 resection should be the primary goal, and a minimally invasive approach should be a secondary goal. The quality of the procedure should not change for the sake of laparoscopy. Laparoscopic colorectal surgery requires adequate training in colorectal cancer surgery and advanced laparoscopy. Surgeons who have completed 50 cases can be considered adequately trained (as SAGES guidelines suggest). The learning curve can be steep and different for each surgeon. Some societies have suggested mentored cases and a minimum yearly volume to achieve good outcomes, as above, because mentorship and proctoring are likely to advance the skill sets required to perform laparoscopic colorectal surgeries. Local/national surgical specialty boards or societies should determine proficiency.

**TABLE 2:**
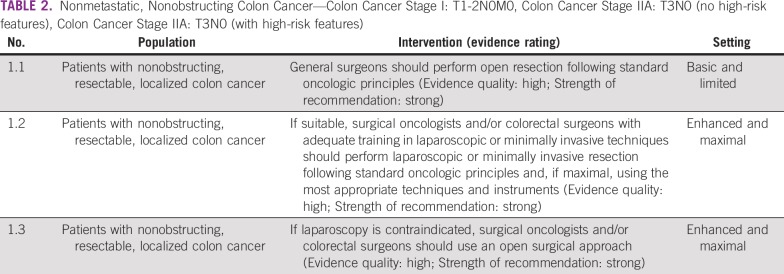
Nonmetastatic, Nonobstructing Colon Cancer—Colon Cancer Stage I: T1-2N0M0, Colon Cancer Stage IIA: T3N0 (no high-risk features), Colon Cancer Stage IIA: T3N0 (with high-risk features)

#### Source guidelines and discussion.

The recommendations on surgery are based on NICE and SAGES recommendations. The NICE guideline was based on a 2006 (subsequently affirmed) evidence review of 19 RCTs and an unpublished individual patient meta-analysis.^16^ As in many systematic reviews, there was heterogeneity in elements, such as time of follow-up.

For settings with a clinician who is a surgical oncologist and/or colorectal surgeon with adequate training in laparoscopic techniques, laparoscopy is preferred because of decreased length of stay in the hospital. Other benefits, with less strong evidence, may include decreased surgical mortality. Risks include anastomotic leakage and conversion to open resection. The provision of the laparoscopic approach is more likely in maximal and enhanced settings. In basic and limited settings, open resection may be more appropriate; both have very similar clinically relevant outcomes according to NICE. The NICE guideline presents both options and found that efficacy outcomes were not statistically significantly different, for example, in three RCTS assessing the risk of mortality during and 30 days after surgery.^16^ It is important to prioritize the goals of surgery. Good quality resection of the tumor is the primary goal. If providers can achieve this with a minimally invasive approach without changing the quality or the nature of the operation, then the patient would benefit from a minimally invasive approach, which the NICE guideline supports (eg, recommendations set 1.2.5) based on evidence reviewed in 2006 and re-reviewed in 2010 in a guidance the main NICE guideline cites.^16^

### Nonobstructing Resectable, Colon Cancer Stages IIB-IIC T4N0

During the informal consensus process, the Expert Panel discussed that patients would benefit more from having an en bloc resection performed in a facility where this was possible rather than having an incomplete (margin-positive or adjacent organs not included) resection at the lower-level facility unless it was an emergency (Table 3).

**TABLE 3:**
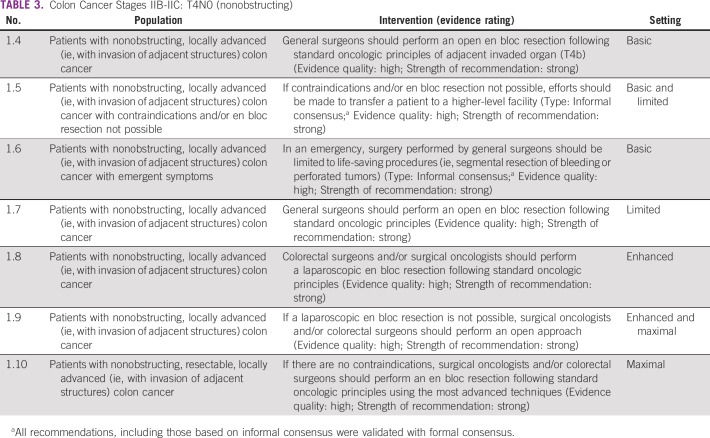
Colon Cancer Stages IIB-IIC: T4N0 (nonobstructing)

#### Source guidelines and discussion.

These recommendations are based on the SAGES and NICE guidelines. SAGES, which focuses on laparoscopy, recommends en bloc surgery for locally advanced adherent colon and rectal tumors (T4b with local extension to structures that cannot be dissected), with weak evidence. There are no RCTs according to SAGES.

Similar surgical principles for locally advanced (nonobstructing T4N0) tumors apply as in early stages of disease, with the primary aim of treatment to remove the tumor with negative resection margins. With locally advanced colon tumors with local invasion of other structures, this guideline recommends an en bloc resection. Clinicians should tailor the surgical approach based on the available expertise and technology. In basic and limited settings, if an en bloc resection to completely remove the malignancy is not possible, patients should be transferred to a facility where this is possible. In more well-resourced settings, the minimally invasive resection is preferred if technically feasible, and there may be a role for additional local-regional therapy (eg, intraoperative radiation) to assist in clearing the margin to reduce the risk of local-regional recurrence. In more technologically advanced settings, this guideline prefers the minimally invasive resection if it is technically feasible.

### Obstructing Colon Cancer Tumors: T3N0 or T4N0 (obstructing)

As with nonobstructing tumors, the main treatment of patients with obstructing colonic lesions is surgery (Table 4). However, when patients present in a clinically obstructed fashion, the surgeon’s decision revolves around whether to perform an emergency surgical resection while still adhering to oncologic principles versus performing a bowel diversion. This latter option is a less time-consuming operation and a temporizing measure, allowing for patient stabilization and further potential work-up and treatment prior to definitive surgery. In the basic and limited resource settings where there are no or limited personnel who have undergone surgical specialization, a general surgeon is responsible for the management of these patients. The general surgeon, therefore, should attempt an emergency resection only if feasible; that is, the surgeon has sufficient expertise managing patients with such advanced disease processes and an en bloc tumor resection can be safely performed, without compromising established oncologic principles. These principles include proximal ligation of the primary arterial supply to the segment harboring the cancer, appropriate proximal and distal margins, and adequate lymphadenectomy.

**TABLE 4:**
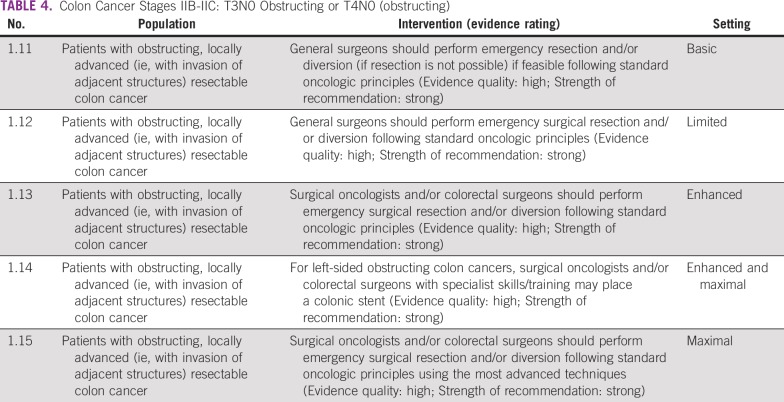
Colon Cancer Stages IIB-IIC: T3N0 Obstructing or T4N0 (obstructing)

In the maximal and enhanced settings, more-advanced local infrastructure and training play a role in surgical decision making. Specialty-trained surgical oncologists and/or colorectal surgeons are more common and available. With this increase in surgical expertise, an en bloc tumor resection should be safely performed without compromising oncologic principles. In the maximal setting, surgeons can perform surgery using the most advanced techniques, such as the laparoscopic approach, provided that they have adequate surgical expertise and oncologic principles are maintained. If resection is not feasible (see oncologic principles in previous paragraph), in addition to the option of diversion, for left-sided lesions, the surgical oncologist or colorectal surgeon may place or request the placement of a colonic stent as a temporizing measure. Although the evidence is weak, stenting may increase the likelihood of completing a one-stage procedure and may decrease the likelihood of an end colostomy.^8^

#### Source guidelines and discussion.

As in the nonobstructing scenario, the goal of surgery is complete removal of the tumor. However, the approach to resection should be tailored to the urgency of the clinical situation, the patient’s condition, the surgeon’s technical ability, and the available infrastructure and/or resources. While there is no specific literature to guide treatment in low-resource settings, these recommendations are based on the NICE, SAGES, and ASCR guidelines, as an obstructing colorectal tumor is a universally encountered problem. The NICE guideline makes specific recommendations regarding obstructing tumors and discusses stents; the guideline also addresses emergency situations. Colonic stents are given as an option in enhanced and maximal settings with appropriately trained and experienced clinicians, with the caveat that high-quality evidence of benefits that outweighs surgery was not available (eg, NICE 1.2.2.4). For example, searches to inform the NICE question on colonic stents 3.2.1 found no direct evidence, even when they looked for the use of stents in palliative or emergency studies. Of the few and low-quality data found, there were no overall survival differences.

For both obstructing tumors and tumors with invasion of other organs, multidisciplinary management is key, and the potential of using chemotherapy to downsize the tumor to make surgical resection more feasible should be discussed by the team.

In basic and limited settings, a temporizing procedure may be most appropriate, whereas in a maximal setting, minimally invasive techniques, such as laparoscopy and robotic surgery, may be attempted insofar as oncologic principles are maintained. Temporizing procedures occur when a patient presents as an emergency (eg, because of the obstruction), but surgeons cannot perform the definitive procedure that needs to be done for various reasons (eg, unable to achieve adequate margins). Instead, a surgery is performed to relieve the obstruction, which provides the provider and patient with time to pursue other avenues (eg, finishing the work-up and exploring the possibility of delivering chemotherapy/radiation).

### High-Risk Obstructing Colon Cancer and Diagnoses Eligible for Adjuvant Treatment

These recommendations are applicable to enhanced and maximal resource settings (Table 5).

**TABLE 5:**
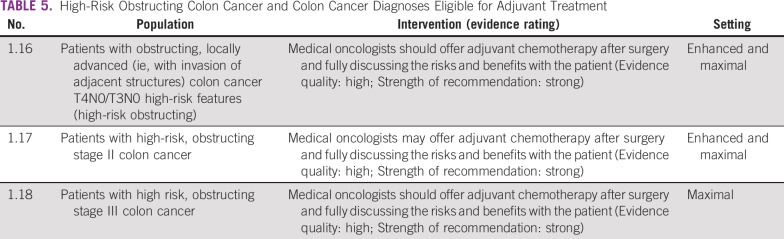
High-Risk Obstructing Colon Cancer and Colon Cancer Diagnoses Eligible for Adjuvant Treatment

#### Source guidelines and discussion.

The recommendations are based on NICE guidelines for patients with high-risk stage II colon cancer in whom adjuvant therapy is “considered.” NICE conducted an evidence review and found limited low-quality data, including pooled data; observational data; and limited RCT data.^15^ An earlier ASCO guideline recommended clinical trials for this population.^17^

There is intermediate-strength data of moderate quality that suggests that patients with high-risk stage II colon cancer have a survival benefit with adjuvant chemotherapy.^15,17^ Although there are several tumor characteristics that are associated with a worse prognosis with stage II disease, there is no standard definition of high-risk stage II colon cancer as defined by the NICE evidence-based review^15^; any one of the following characteristics would be considered high risk: extramural vascular invasion, grade 3/poorly differentiated tumors, T4 stage/perforation, obstructive tumors, mucinous tumors, fewer than 12 lymph nodes harvested, and tumor budding. Because of the lack of data directly addressing the value of adjuvant therapy in patients with high-risk stage II colon cancer and the modest effect of adjuvant therapy in patients with standard-risk stage II disease, we suggest that patients with microsatellite stable/mismatch repair–proficient high-risk stage II colon cancer may receive adjuvant chemotherapy in the advanced and maximal settings after discussion of the risks and benefits with the patient.^15^ In these situations, mismatch repair or microsatellite instability assessment is necessary (where available) because it may influence decisions on adjuvant therapy, particularly for patients with stage II colon cancer. For more information on biomarkers in GI cancer, see the American Society for Clinical Pathology, College of American Pathologists, Association for Molecular Pathology, ASCO guideline on molecular biomarkers for the evaluation of colorectal cancer.^18^

Patients with stage III colon cancer should receive adjuvant chemotherapy in the enhanced and maximal settings based on the NICE recommendation for adjuvant chemotherapy for patients with stage III colon cancer. While the NICE guideline recommended specific agents (eg, fluoropyrimidines, based on 2006 recommendations), there is more recent evidence, from studies suggested by Panel members rather than found by systematic review, that the adjuvant therapy for patients with stage III include a combination chemotherapy regimen of fluoropyrimidine and oxaliplatin.^19-22^ More recent data have examined the duration of adjuvant therapy, suggesting that for some tumors (eg, T3, N1), 3 months of combination therapy, specifically with the combination of capecitabine and oxaliplatin, may be equivalent to 6 months in the adjuvant setting; however, this question is not yet settled.^19^ It is outside the scope of this resource-stratified guideline to review the comparative chemotherapy agent data (eg, capecitabine *v* fluorouracil, 3 *v* 6 months), and clinicians in resource-constrained settings should be guided by national availability of agents, cost/patient finances, and clinician expertise in managing the administration and adverse effects of these agents regarding available effective agents. It is however recognized that there are significant ramifications of this discussion in basic and limited settings. For example, although it may be much easier and cost effective to receive 3 months of therapy that includes an oral medication than an alternative regimen for 6 months, the cost of the drug may not be equivalent, and thus, the decision to receive adjuvant therapy, which regimen, and what duration needs to be weighed against region-specific feasibility. The resources for the treatment of patients with stage III colon cancer are primarily in the maximal and enhanced settings; however, because this guideline does recommend further treatment in these settings, clinicians may explore using them in basic and limited settings as well. The caveats are availability of the medication and expertise in administrating the medication (oncologist or other trained medical provider). The costs involved may require modifying the treatment regimen.

### Clinical Question 2

What is the optimal treatment of patients with rectal cancer stages I-III?

Surgery is the primary treatment modality of early rectal cancer. Following the principle of total mesorectal excision (TME) is critical to achieve a good outcome in patients with rectal cancer. In basic and limited resource settings, this may need to be done with the best expertise available in that setting. Local general surgeons may do this if they understand and have been trained in TME. Local excision may be appropriate in certain situations. However, basic and limited local settings may not have the necessary infrastructure and expertise to select patients for local excision. In enhanced and maximal settings, the guideline recommends a surgeon with training and expertise in rectal cancer surgery. If the technology and expertise are available, clinicians may pursue minimally invasive approaches in these settings (Tables 6, 7, and 8).

**TABLE 6:**
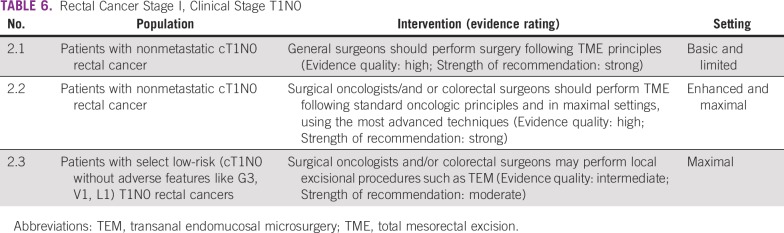
Rectal Cancer Stage I, Clinical Stage T1N0

**TABLE 7:**
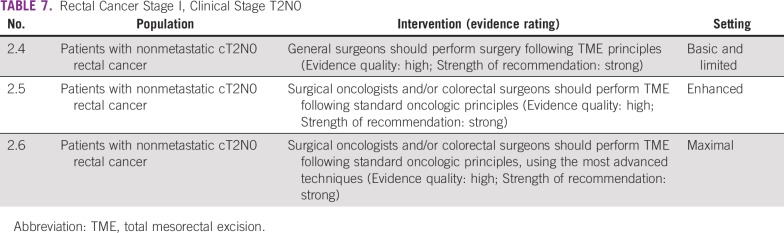
Rectal Cancer Stage I, Clinical Stage T2N0

**TABLE 8:**
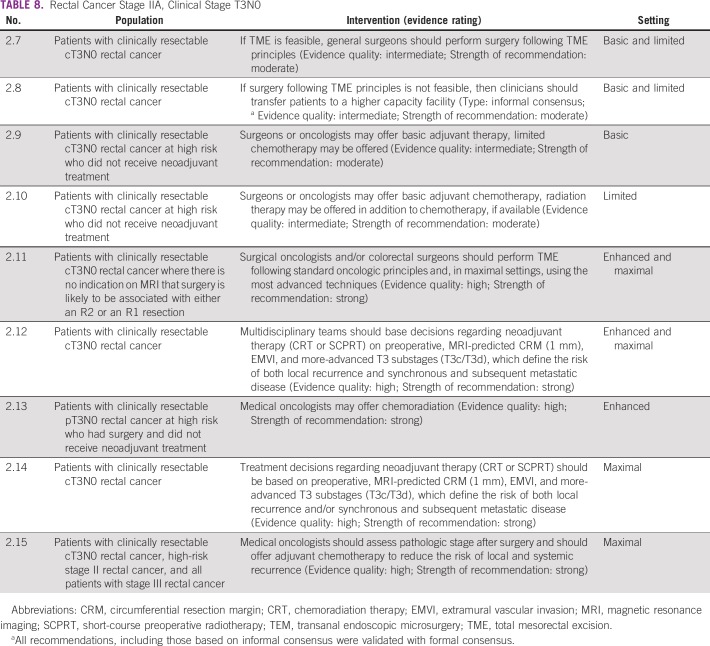
Rectal Cancer Stage IIA, Clinical Stage T3N0

#### Source guidelines and discussion.

These recommendations are largely based on the ASCR and ESMO guidelines. The ASCR surgical recommendations reflect a range of quality of evidence, including lack of RCT evidence; however, they rated the TME recommendation as high quality. For laparoscopy, they note that there is less high-quality evidence than for patients with colon cancer.^9^ The ASCR laparoscopic TME recommendation was based on two RCTs, a meta-analysis, and some (presumably observational) prospective trials, and the authors noted the need for more RCT results; the ESMO guideline does not explicitly recommend laparoscopic surgery. The evidence is not fleshed out in the ASCR guideline for TME (in recommendation Radical Excision 2). The ESMO transanal endomucosal microsurgery (TEM) and TME recommendations were based on prospective cohort studies, and they rated them both IIIA (ie, prospective cohort studies graded as strong evidence and strong recommendation [eg, ASCO’s recommendation 2.1 corresponded with the ESMO recommendation for “local excisional procedures such as TEM are appropriate as a single modality for early cancers (cT1N0 without adverse features like G3, V1, L1) [III, A].)”]^12^^[p6]^).

### Chemotherapy and Radiation

Recommendations on chemotherapy and radiation start at 2.9 (Table 8). An important aspect of the management of rectal cancer is to limit the risk of local-regional recurrence in the pelvis. For patients with tumors at increased risk of local-regional recurrence in the pelvis, preoperative chemotherapy with radiation is recommended. Because clinical staging of rectal cancer requires radiographic and technical skill, for patients with tumors that may be considered high risk for local-regional recurrence (indeed, most rectal tumors that are diagnosed), multidisciplinary management is recommended. Neoadjuvant chemoradiation is recommended for most patients with cT3N0 and stage III rectal cancers in the enhanced and maximal resource settings. Short-course neoadjuvant radiation may be discussed based on two random assignment trials.^23,24^ Surgery by the TME approach has been proven to minimize the risk of local-regional recurrence. These good-risk cT3 rectal tumors are defined by the ESMO 2013 evidence-based guideline as follows: cT3a (< 1-mm invasion into the subserosa) without involvement of the mesorectal fascia and higher in the rectum (above the levator muscles). When all of these features of the tumor are met, TME surgery without neoadjuvant chemoradiation is recommended.

However, radiation therapy and/or advanced imaging, such as magnetic resonance imaging, is not available or has limited availability in basic and limited settings.^25^ In this case, NACT alone (without neoadjuvant chemoradiation) is not recommended due to a lack of evidence. This recommendation will be revisited depending on emerging evidence. Advanced radiographic imaging may also not be available either, thereby limiting the ability to distinguish between low- and high-risk rectal cancer. Consequently, patients and clinicians in these settings should proceed to TME resection if imaging is available to determine resectability. The Expert Panel notes that surgical pathology evaluation may upstage some patients (more extensive local invasion or nodal involvement), and this may inform treatment decisions regarding adjuvant therapy. Following resection, patients who have pathologic higher-risk disease should receive adjuvant chemotherapy preferably with radiation, if available.

Potential benefits of neoadjuvant therapy include lowering the risk of local recurrence.^9^ Potential risks include sexual morbidity and GI adverse events. When TME is not feasible (eg, due to clinical factors or surgeon availability), the role of chemotherapy with radiation in reducing the risk of local-regional recurrence is greater. For all patients who have undergone NACT with radiation therapy, postoperative chemotherapy is recommended,^12^ extrapolating largely from the colon cancer adjuvant data. A full discussion of adjuvant therapy following neoadjuvant chemoradiation and surgery for rectal cancer is beyond the scope of this resource-stratified guideline.

#### Source guidelines and discussion.

These recommendations on neoadjuvant and/or adjuvant therapy are largely based on the ASCR and ESMO guidelines. The neoadjuvant therapy recommendations are based on ASCR (which it rated as a strong recommendation with high-quality evidence IA), citing two trials on radiation and one on chemoradiation, one small comparison of the two approaches, and one trial comparing neoadjuvant and adjuvant chemoradiation (indicating lower local recurrence with neoadjuvant therapy); some were phase II trials. Therefore, ASCO would not likely call this high-quality evidence in a non–resource-stratified guideline. ASCR cites two Cochrane reviews on neoadjuvant therapy, so perhaps one could say that there is insufficient evidence on optimal sequencing for neoadjuvant therapy. The ESMO guideline states that “NACT alone is not recommended for the treatment of localised, non-metastatic disease outside clinical trials”^12^^(page iv31)^ because there was lower evidence of benefits outweighing risks.

For adjuvant chemoradiation, ASCR rates this 1B (a strong recommendation with moderate-quality evidence) and cites several RCTs, albeit commenting that they have limitations. For chemotherapy alone for patients who received neoadjuvant therapy, ASCR rates its recommendation 1A but does not review the evidence. The ESMO guideline states that when patients have not received neoadjuvant radiation therapy and have a high risk of recurrence, adjuvant chemoradiation is an option in selected cases, depending on postsurgical tests (with 1A rating). ESMO refers to trials and meta-analyses as evidence.

### Clinical Question 3

What are the optimal strategies for post-treatment surveillance for patients treated for early colorectal cancer?

The recommendations for follow-up for colon cancer are listed in Table 9 and for rectal cancer in Table 10; Tables 11 and 12 list summaries of the full follow-up recommendations.

**TABLE 9:**
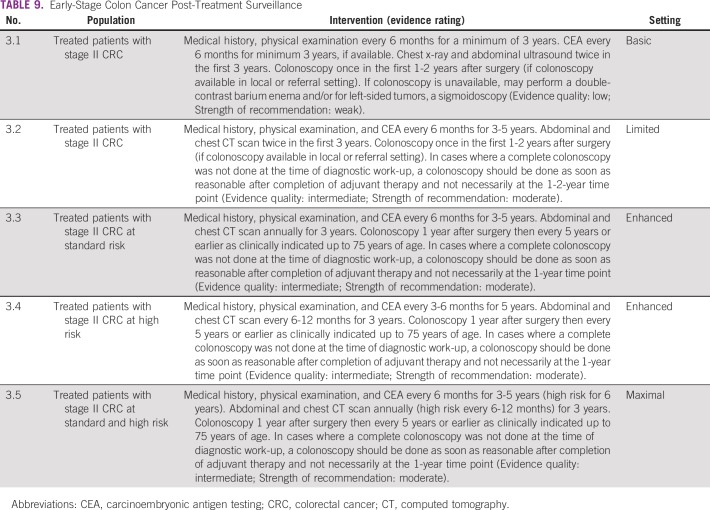
Early-Stage Colon Cancer Post-Treatment Surveillance

**TABLE 10:**
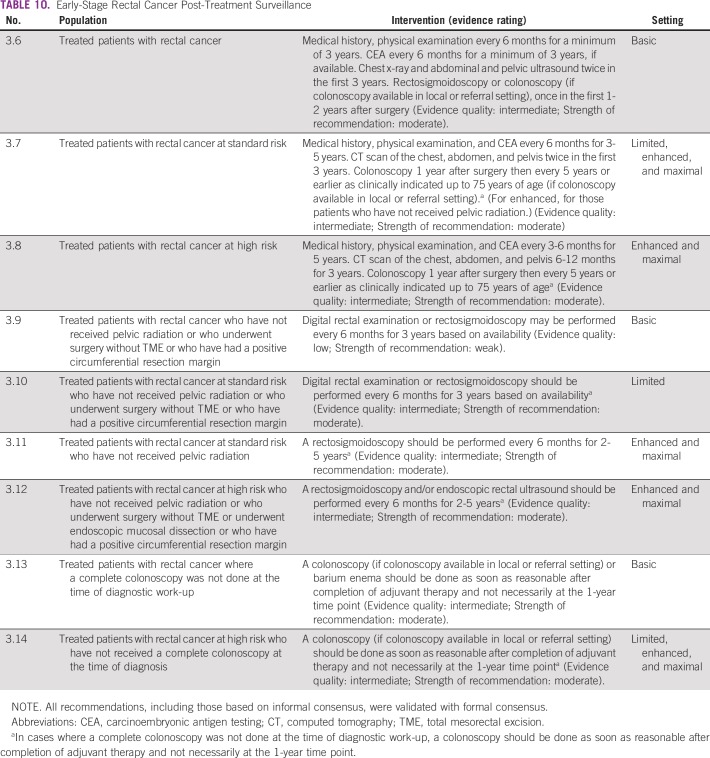
Early-Stage Rectal Cancer Post-Treatment Surveillance

**TABLE 11:**
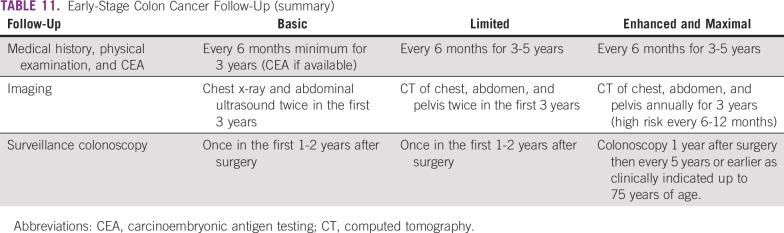
Early-Stage Colon Cancer Follow-Up (summary)

**TABLE 12:**
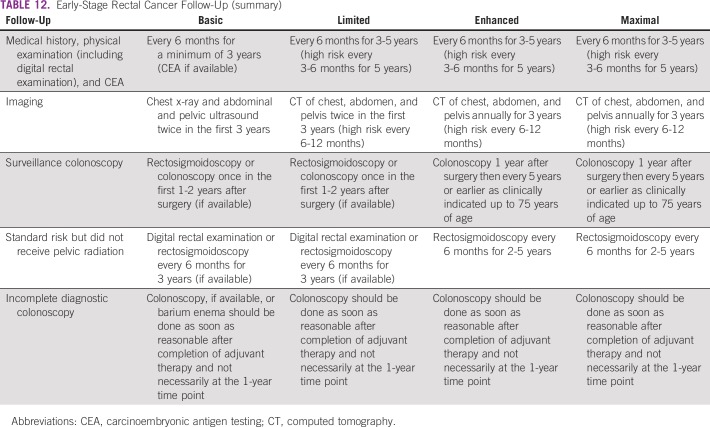
Early-Stage Rectal Cancer Follow-Up (summary)

#### Source guidelines and discussion.

There are multiple existing guidelines from maximal resource settings on follow-up. The recommendations that were adapted here, primarily to provide guidance to limited and basic settings, are based on the 2013 ASCO guideline endorsement of the CCO follow-up surveillance and secondary prevention (one recommendation was also based on NICE). This endorsement was for patients with stage II and III colorectal cancer. There is a paucity of data on follow-up for patients with stage I colorectal cancer. In this resource-stratified guideline, therefore, the recommendations for the follow-up of patients with stage I were based on expert consensus due to the lack of evidence on this topic. The original CCO guideline was based on 11 other guidelines. The population covered was focused on survivors of stage II and III colorectal cancer. The recommendations for basic and limited settings are based on the probable lack of colonoscopy. In cases where a complete colonoscopy was not done at the time of diagnostic work-up, a colonoscopy should be done as soon as reasonable after completion of adjuvant therapy and not necessarily at the 1- to 2-year time point. In the basic setting, if colonoscopy is unavailable, clinicians may perform a double-contrast barium enema and/or for left-sided tumors, a sigmoidoscopy.

## SPECIAL COMMENTARY

### Cultural Context and Age

In the context of these guidelines, the Expert Panel recognizes that because these guidelines are applicable in many different cultures, cultural sensitivities are particularly important when communicating with patients and families regarding decisions based on either chronologic or functional age. It is not within the scope of this guideline to give this discussion the space it deserves, and the reader is referred to ASCO’s Practical Assessment and Management of Vulnerabilities in Older Patients Receiving Chemotherapy guideline; ASCO’s resource-stratified practice guideline on palliative care, specifically regarding spiritual assessment; and ASCO’s Patient-Clinician Communication guideline.^26-28^

Although this guideline recommends chemotherapy regardless of age, age can be a factor in clinician-patient decision making. Chronologic age is mentioned in the follow-up recommendations based on existing guidelines. However, chronologic age may not be sufficient for decision making, and the Expert Panel encourages clinicians to use functional age. The Expert Panel would like to emphasize that life expectancy and underlying health status are important to assess and take into consideration in discussions with family and caregivers regarding chemotherapy. ASCO’s Practical Assessment and Management of Vulnerabilities in Older Patients Receiving Chemotherapy guideline states, “In patients ≥ 65 years receiving chemotherapy, geriatric assessment (GA) should be used to identify vulnerabilities that are not routinely captured in oncology assessments,”^26^^(p2326)^ as well as to assess life expectancy.^26^

## COST IMPLICATIONS

An ASCO literature search focusing on high-quality systematic reviews of published cost-effectiveness analyses in low-resource settings was conducted, and none were found. The Guideline Panel identifies the need for cost-effective analyses of the treatment of patients with early-stage colorectal cancer from low-resource settings.

## LIMITATIONS OF THE RESEARCH AND FUTURE DIRECTIONS

There were limitations of the evidence to inform some of the recommendations. There were limited published data on:

- Most studies were conducted with populations in high-resource settings.
- Treatment of patients with nonobstructing colon cancer stages IIB-IIC (T4N0), including the role for additional local-regional therapy
- Weak evidence on stenting of patients with colon cancer stages IIB-IIC obstructing
- Specific literature to guide treatment in low-resource settings of patients with colon cancer stages IIB-IIC: T4N0 obstructing or T3N0 obstructing
- Value of adjuvant therapy in patients with high-risk stage II colon cancer
- Treatment of patients with rectal cancer stages I-III, especially with laparoscopic approaches
- Refining duration of adjuvant therapy
- Cost-effectiveness research and modeling

Refining adjuvant therapy is an ongoing research effort. For example, the large IDEA study^19^ suggests that in some patients, 3 months of adjuvant therapy may be adequate. There is a large US intergroup study examining the role of radiation for more proximal rectal tumors (eg, farther from the anal verge; PROSPECT study [ClinicalTrials.gov identifier: NCT01515787]). Further studies on the role of total neoadjuvant therapy are being developed, bolstered by the high pathologic complete response rates in phase II studies.^29^ These studies will help to define further the adjuvant treatment of localized colorectal cancer. In addition, future studies that tailor therapy based on molecular profiles of tumors may further refine our treatments.

Therefore, the Expert Panel suggests that research, especially RCTs, be conducted and/or completed on the topics for which there are currently insufficient data.

ASCO believes that cancer and cancer prevention clinical trials are vital to inform medical decisions and improve cancer care and that all patients should have the opportunity to participate.

### ADDITIONAL RESOURCES

Additional Information including data supplements, evidence tables, and clinical tools and resources can be found at www.asco.org/resource-stratified-guidelines. Patient information is available there and at www.cancer.net.

**Related ASCO Guidelines**

- Palliative Care in the Global Setting: ASCO Resource-Stratified Practice Guideline (http://ascopubs.org/doi/10.1200/JGO.18.00026)^27^
- Patient-Clinician Communication: American Society of Clinical Oncology Consensus Guideline (http://ascopubs.org/doi/10.1200/JCO.2017.75.2311)^28^
- Early Detection for Colorectal Cancer: ASCO Resource-Stratified Practice Guideline (http://ascopubs.org/doi/10.1200/JGO.18.00213)

## Figures and Tables

**TABLE A1:**
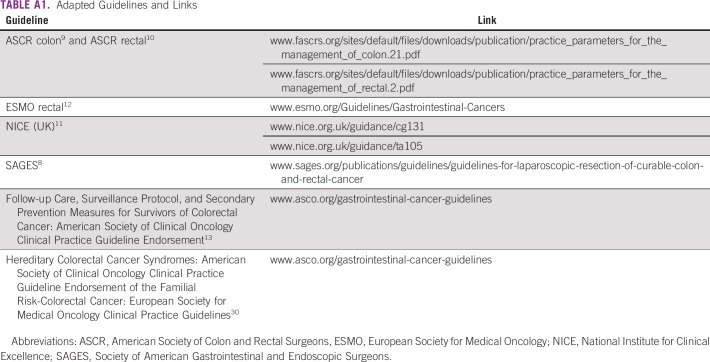
Adapted Guidelines and Links

**TABLE A2:**
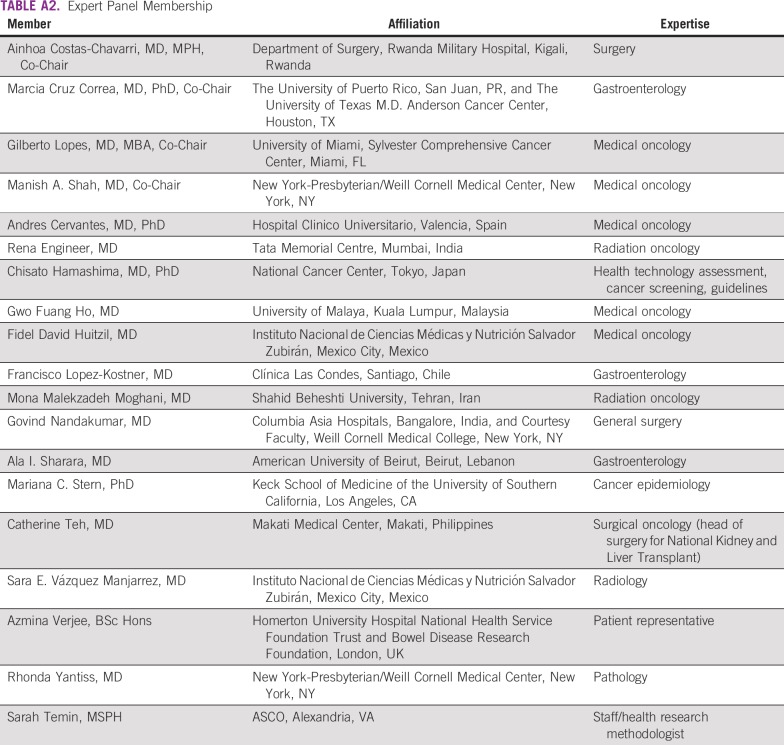
Expert Panel Membership

## Data Availability

The following represents disclosure information provided by authors of this manuscript. All relationships are considered compensated. Relationships are self-held unless noted. I = Immediate Family Member, Inst = My Institution. Relationships may not relate to the subject matter of this manuscript. For more information about ASCO's conflict of interest policy, please refer to www.asco.org/rwc or ascopubs.org/jgo/site/misc/authors.html. **Speakers’ Bureau:** Medtronic **Consulting or Advisory Role:** Pfizer **Research Funding:** Merck Sharp & Dohme (Inst), EMD Serono (Inst), AstraZeneca (Inst), AstraZeneca, Blueprint Medicines (Inst), TESARO (Inst), Bavarian Nordic (Inst), Novartis (Inst), G1 Therapeutics (Inst) **Consulting or Advisory Role:** Merck Serono, Bayer AG, Roche, Genentech, Eli Lilly, Amgen, Servier, Novartis, Takeda Pharmaceuticals **Speakers’ Bureau:** Merck Serono, Roche, Servier **Research Funding:** Roche (Inst), Merck Serono (Inst), Servier (Inst), Novartis (Inst), Eli Lilly (Inst), TESARO (Inst), Johnson & Johnson (Inst), MedImmune (Inst), Theradex Systems (Inst) **Travel, Accommodations, Expenses:** Roche **Patents, Royalties, Other Intellectual Property:** Licensing agreement with Johns Hopkins University on technology **Honoraria:** Pfizer, AstraZeneca, Merck, Eli Lilly, Merck Sharp & Dohme, Novartis **Consulting or Advisory Role:** AstraZeneca, Pfizer, Boehringer Ingelheim, Novartis, Merck Sharp & Dohme, Roche, Genentech, The Menarini Group **Speakers’ Bureau:** Boehringer Ingelheim, Celgene, Merck, Eli Lilly **Research Funding:** Merck Serono, Merck Sharp & Dohme, Samsung Bioepis, Tessa Therapeutics, AB Science, Pfizer, Eli Lilly **Travel, Accommodations, Expenses:** Eisai, Boehringer Ingelheim, AstraZeneca, Merck Sharp & Dohme, Pfizer, Novartis **Consulting or Advisory Role:** Roche **Speakers’ Bureau:** Eli Lilly **Research Funding:** Eli Lilly (Inst), Bristol-Myers Squibb (Inst) **Patents, Royalties, Other Intellectual Property:** Principal investigator for a gastric cancer trial (Inst) **Other Relationship:** Bristol-Myers Squibb **Consulting or Advisory Role:** Novartis **Travel, Accommodations, Expenses:** Merck, Roche **Honoraria:** AbbVie, Janssen-Cilag, AstraZeneca, Takeda Pharmaceuticals, MSD, Pfizer (I), MSD (I) **Consulting or Advisory Role:** MSD, Takeda Pharmaceuticals, Gilead Sciences **Research Funding:** AbbVie, Gilead Sciences, Janssen-Cilag, Takeda Pharmaceuticals, Astellas Pharma **Travel, Accommodations, Expenses:** Takeda Pharmaceuticals, Pfizer (I), AbbVie, MSD, MSD (I), Janssen-Cilag **Patents, Royalties, Other Intellectual Property:** Joint patent with the University of Southern California and Illumina for the discovery of a gene expression classifier that predicts recurrence of prostate cancer among men who underwent radical prostatectomy (Inst) **Consulting or Advisory Role:** Astellas Pharma **Research Funding:** Gilead Sciences (Inst), Merck (Inst), Boston Biomedical (Inst), Oncolys BioPharma (Inst) No other potential conflicts of interest were reported
